# A Case of Actinomycosis

**Published:** 1907-09-15

**Authors:** George Zederbaum

**Affiliations:** Charlotte, Mich.


### A CASE OF ACTINOMYCOSIS.

BY GEORGE ZEDERBAUM, D.D.S., CHARLOTTE, MICH.

Read before the Southwestern Michigan Dental Society, at Battle Creek, April 9, 1907.

I am here today to present to you something out of the ordinary line of dental society meetings. In selecting for my subject the description and the treatment of a case of actinomycosis that came into my hands. My particular subject is within these very domains and is concerned with the oral surgery branch of our profession, chiefly. In presenting this subject of "Actinomycosis," I wish to show the necessity for every general practitioner of dentistry being familiar with the requirements along the oral surgery line; to show that we as should be able to take care of most oral diseases. I also contend that we ought to be in position to make our brother, the general practitioner of medicine, understand that we are not merely skilled mechanics, but that we are skilled professional men as well, and have studied anatomy, physiology, bacteriology materia medica, oral surgery, etc., sufficienty to be able to intelligently care for the usual case that presents. And that is within the legitimate bounds of a dental practitioner, unless it be a case for a major operation, when a specialist or a consulting surgeon would be desirable to undertake treatment.

Actynomycoses has often been mistaken for osteosarcoma in regions where bones were involved and microscopical examinations were necessary to determine a correct diagnosis. Dr. Bevan, of Rush Medical College, and an authority on this particular subject says: "Clinically, actinomycosis appears under four different forms, from four different routes of infection: 1. Head and neck actinomycosis, with infection from mouth and pharynx. 2. Chest actinomycosis, through the respiratory tract. 3. Abdominal actinomycosis, with infection probably always through the alimentary canal; possibly through the genital tract of the female. 4. Actinomycosis of the skin. The frequency of occurrence of the disease in these various regions is found to be, roughly speaking, about 50 per cent of head and neck actinomycosis, from 15 to 20 per cent of chest cases, from 20 to 25 per cent of abdominal cases, about 2 per cent of skin cases and a small balance where it is difficult to determine the route of infection." We, as dentists, are concerned chiefly with the first division of the above classification, which, you will note, supplies half of the total number of such cases. The common names are "wen" and more often "lumpy-jaw." The glands of the throat are usually swollen to extreme; appetite, naturally, becomes poor, tongue irritated, saliva profuse and a peculiar odor of b eath is always noticed. This has been observed in horses, in oxen and in man, and is known to be caused by the attack of vegetable parasites known by the name of "actinomyces," or "ray fungus." The parasite gets into some wound or slight abrasion of the mucosa, and when in the maxillary region of man, usually by way of pyorrheal pockets. The parasite, it is particularly said, has its resting place on barley-straw, and the sharp barley awns are sometimes blamed for causing the preliminary abrasions by which the parasite gains access into submucosal tissues of a herbivorous animal. The ray fungus is also common in the air, water and soil, and especially as a rust on various grains and grasses; therefore, it is not uncommon to find the typical actinomycosis cases among the farming class. The ravages of this disease, we see, are mostly in connection with the lower jaw bones and the glands of the throat and submaxillary space. These parts become swollen and very hard. In many cases the tongue becomes so large that suffocation is a well-pronounced feature. Finally, the hard glands suppurate and the purulent, earthy-smelling discharge contains sufficient quantity of actinomyces to make it feel quite gritty. We may stain a little of the pus with methylene-blue, or still better by carbolfuchsin or even the Gram method and examine the slide microscopically. The ray or club-shaped rods of the actinomycosis fungus can be readily discovered. These parasites cannot be mistaken. They look like a number of clubs or rods arranged in the form of a star radiating from a common center, and it is from this shape that it is known as the ray-fungus. Potassium iodid and mercury biniodid, and the late Dr. Bevan's treatment with copper sulphate, are among the well-known specifics. The administration of the two first mentioned drugs has to be done with great care, preferably intermittently, or other systemic disturbances will follow. The disease should be recognized early and specifics administered from the start. Death, when the disease is in the region of the mouth, usually results from starvation or suffocation. Prognosis in most lumpy-jaw cases is favorable if proper treatment and surgical interference are commenced early. In other regions not so favorable and in pyemic forms entirely hopeless.

Recently the following case came under my observation: Early in September a man, aged 44, and of good physique, was brought in by an old patient for examination and advice. The history of the case was simple; There was considerable swelling at the angle on the right side of the lower jaw, extending anteriorly and inferiorly. No pain at any point after a scrutinizing digital examination of the parts. Within the preceding week, when the trouble was first noticed, the patient had three lower right molars extracted, but swelling did not decrease. The mouth could not be easily opened. The patient complained of lack of appetite, sleeplessness, and difficulty in breathing. Facial color was bad, circulation poor and lips almost cyanosed. On examining the mouth it was found to be in a very uncleanly condition; extensive pyorrhea present, some pockets quite deep and discharging; odor very fetid. Upper right molar had a putrescent pulp and was beyond crowning. Other teeth fairly good. Patient gave satisfactory history as to health and habits and his occupation as that of a farmer. Family history failed to show any carcinomatous, sarcomatous or tuberculous possibility. Acting upon my advice, the mouth and teeth were cleansed in the best possible manner, and the upper right molar mentioned was extracted, patient taking a general anesthetic. I gave prescription for a 10 per cent solution of iodopetrogen to be used locally three times daily, rubbing it in carefully over the whole affected area; ordered a thick anti-phlogistine compress applied at night and gave glyco-thymoline mouth wash to be used ad libitum. Advised also one ounce of magnesium sulphate and to remain indoors and keep me informed.

On the third day after this first visit, he came again. Swelling had extended to include the submaxillary, superior and inferior carotid and the anterior portion of the subclavian triangles. Induration was pronounced at the hyoid area, emitting a species of wooden sound upon palpitation. On digital pressure upon the two of the most dependent points there was tenderness; one directly above and to the right of the hyoid bone, and the other immediately below the right angle of the mandible. Temperature (sublingual) 100 degrees, breath still fetid and tongue badly coated and somewhat swollen at the base. Upon further questioning I learned that, prior to the commencement of his present trouble, he had been threshing wheat and doing general harvesting work about the field. There were no additional symptoms present at this second examination. Pus formation was progressing beyond a doubt and flaxseed poultices were ordered from now on, also small oft-repeated doses of calomel.

On the following day, I visited my man at his residence. Temperature 102 degrees. He was very restless during the night, and, as the swelling and the induration were rapidly getting worse, he had fear of choking to death. I injected ten drops of echafolta into the anterior, the larger of the two pronounced points, and left antikamnia and codein tablets with instruction to take one every two hours; gave echafolta solution for internal administration and ordered continuation of poultices. Upon returning to the office, I opened my oral surgery text-book and also the American System of Surgery and read over the chapters pertaining to diagnosis and treatment of actinomycosis. In case just described all the clinical signs were present, and I decided that this was the disease I had to deal with, and that the only verification necessary would be the examination of pus, of which I will speak later.

At 2:30 the next morning, I was called by telephone by patient's daughter, who, in excited tones, said that her father was choking to death and that I must get there at once. Remembering the condition of my patient when I saw him some twelve hours previous, I did not doubt such a possibility; especially so after having on the previous day consulted two veterinaries, who explained that such might be the culmination of a lumpy jaw attack. I hurried to the residence with oral surgery instruments, also chloroform, etc., and when within a few rods of destination met the family physician, who was called simultaneously. I hurriedly explained the situation before we entered and readily saw that this worthy doctor did not approve of a dentist handling such a case. However, I was called and had charge of the case and was not going to back out. The probability was that I knew just about as much about this particular case as he did. The patient was frightened to a great degree, pacing to and fro and groaning. Swelling was more pronounced and the two points before mentioned very tender. The skin over the whole affected area was thrown into the characteristic folds, or rugae, if you please, of actinomycosis. Upon inserting aspirating needle I succeeded in drawing some pus and made several slides. None of these showed the ray fungus. The echafolta solution I I injected on previous day had probably excluded these fungi to a great degree. The doctor, however, corroborated my diagnosis, and we decided to work together. We placed the patient on potassium iodid, ten grains, three times daily, gradually increasing to 60 grains per diem, and urged vigorous use of poultices. Within two more days I opened the most dependent point of the lower swelling and explored the numerous sinuses, reaching with probe to the angle of the jaw. About a pint of most virulent, peculiarly foul-smelling pus was drained. The characteristic yellowish-gray granules of actinomycosis were present, thus conclusively corroborating our diagnosis. The wound was irrigated with bichlorid solution and lightly packed with iodoform gauze. I made several more slides, and after staining with methylene-blue only one revealed actinomyces, while all showed the giant cells. Irrigating and dressing were done twice daily for a time and eventually once each day. Wound healed entirely within ten days; in fact, it was a hard matter to prevent it from healing too fast. At the end of ten days patient came to office and I noticed a dormant sinus partially filled with pus immediately below the first opening. I lanced the same and washed out with potassium permanganate solution. This also healed very shortly. Patient returned to work and has been gaining ever since. Induration about the hyoid area persisted for many weeks. Upon digital lateral movement of the bone, a distinct crepital sound was made, somewhat alarming me for a time. Massaging carefully but persistently caused this condition to disappear at the end of several weeks. The patient reported regularly for prophylactic treatment and is now entirely out of danger.

I wish to add here a few words explanatory of my stand relative to my friend, the doctor, who called at my office one day, after the case was well in hand, and very patronizingly assured me that he would not attempt to make me any trouble, but that there was no question in his mind that I, only a D. D. S., had nothing to do with such a case but turn it over to the physician. I argued with him for half an hour or more, explained that I was obliged to take and pass an examination in oral surgery, that the course I took from one of the leading schools embodied in its curriculum two years of that branch; that I took State Board examination and had to answer among others, a question on diagnosis and treatment of actinomycosis. But, no, nothing would convince him that I acted within my jurisdiction and within my knowledge. I was determined to prove my point and immediately wrote Dr. A. L. LeGro, then Secretary of the State Board, and, also, to Dr. Oakman, now President of same Board, asking whether I had in any way overstepped my boundary as a D. D. S. in treating this case of actinomycosis. Their replies showed that I not only had a perfect right to take such a case, but that I and every other dental practitioner should know how to take care of a patient suffering from actinomycosis, both surgically and medicinally. I am sure, gentlemen, that since my friend the doctor has read these letters, he has a better understanding of the relations that should exist between the two professions.

We know, of course, that the field of the dentist and the general practitioner or specialist in medicine often overlap, and this is one of the many illustrations of the fact. We, as dentists, must keep well abreast of the times in the medical and surgical as well as the mechanical science of our profession; and, what is still more important, apply this knowledge to practical use, for then and only then will we be classed among the progressive specialists of medicine.

Oral surgery, then, makes the same demand upon our skill and intelligence as any other branch of our profession. These demands are, however, not excessive or in any way unreasonable, and we, as units, representing a noble branch of medical science, ought to be able to meet them now and always.

While I will admit that I recognized the case I described, simply by accident, I assure you that a second case of like nature would be recognized and treated because of the fuller knowledge of it; and I believe that should any of you have such a case, you will certainly congratulate yourselves that you were here today. It is often hard to find a characteristic specimen of ray fungus, as the organism easily undergoes a degenerating process. The treatment of copper sulphate, as outlined by Dr. Bevan and Dr. Post of Rush Medical College, is based on sound theory. Most of you, no doubt, have read that progressive agriculturists have been trying long and faithfully to find an effective but harmless medicinal agent that would exterminate the ray fungus, which is so abundant in air, water, soil, that it attacks fruits, grains and grasses. The copper sulphate, in weak solutions, proved equal to the test, and is now used exclusively for that purpose. The treatment of actinomycosis with copper sulphate is very successful, even in some cases of the abdominal variety, where the older specific proved valueless. We cannot send all our unusual cases to specialists.

